# Comparative assessment of range‐wide patterns of genetic diversity and structure with SNPs and microsatellites: A case study with Iberian amphibians

**DOI:** 10.1002/ece3.6670

**Published:** 2020-09-15

**Authors:** Miguel Camacho‐Sanchez, Guillermo Velo‐Antón, Jeffrey O. Hanson, Ana Veríssimo, Íñigo Martínez‐Solano, Adam Marques, Craig Moritz, Sílvia B. Carvalho

**Affiliations:** ^1^ CIBIO/InBIO Centro de Investigação em Biodiversidade e Recursos Genéticos da Universidade do Porto Vairão Portugal; ^2^ Museo Nacional de Ciencias Naturales CSIC Madrid Spain; ^3^ Centre for Biodiversity Analysis and Research School of Biology The Australian National University Canberra ACT Australia

**Keywords:** DArTseq, *Hyla molleri*, Iberian Peninsula, *Pelobatescultripes*, population genetics, microsatellites

## Abstract

Reduced representation genome sequencing has popularized the application of single nucleotide polymorphisms (SNPs) to address evolutionary and conservation questions in nonmodel organisms. Patterns of genetic structure and diversity based on SNPs often diverge from those obtained with microsatellites to different degrees, but few studies have explicitly compared their performance under similar sampling regimes in a shared analytical framework. We compared range‐wide patterns of genetic structure and diversity in two amphibians endemic to the Iberian Peninsula: *Hyla molleri* and *Pelobates cultripes,* based on microsatellite (18 and 14 loci) and SNP (15,412 and 33,140 loci) datasets of comparable sample size and spatial extent. Model‐based clustering analyses with STRUCTURE revealed minor differences in genetic structure between marker types, but inconsistent values of the optimal number of populations (K) inferred. SNPs yielded more repeatable and less admixed ancestries with increasing K compared to microsatellites. Genetic diversity was weakly correlated between marker types, with SNPs providing a better representation of southern refugia and of gradients of genetic diversity congruent with the demographic history of both species. Our results suggest that the larger number of loci in a SNP dataset can provide more reliable inferences of patterns of genetic structure and diversity than a typical microsatellite dataset, at least at the spatial and temporal scales investigated.

## Graphical abstract

We compared patterns of genetic structure and diversity between microsatellites and SNPs in two amphibians from the Iberian Peninsula: *Hyla molleri* and *Pelobates cultripes*. Larger number of loci from SNP dataset can provide more reliable inferences of patterns of genetic structure and diversity than a typical microsatellite dataset (photo: *Pelobates cultripes*; credits: Guillermo Velo‐Antón).

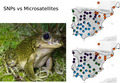

## 1 INTRODUCTION

Nuclear microsatellites became popular during the 1990s as a powerful tool to assess patterns of genetic variation in populations (Allendorf, 2017; Ellegren, 2004). While they are still widely used, the development of Genotyping‐by‐Sequencing techniques, like RADseq (Baird et al., 2008; Miller, Dunham, Amores, Cresko, & Johnson, 2007) and similar techniques of genome complexity reduction (e.g., ddRAD and bestRAD), coupled with the decreasing costs of massive parallel sequencing, have extended the reach of massive single nucleotide polymorphism (SNP) genotyping to the study of nonmodel organisms (Allendorf, 2017; Andrews, Good, Miller, Luikart, & Hohenlohe, 2016; Baird et al., 2008; Davey et al., 2011; Peterson, Weber, Kay, Fisher, & Hoekstra, 2012; Putman & Carbone, 2014). This has led to a discussion about the relative benefits of using each type of marker in conservation and evolutionary biology (Allendorf, 2017; Hodel et al., 2017; Morin, Luikart, & Wayne, 2004; Puckett, 2017).

Mutation rates in microsatellites are several orders of magnitude higher than those estimated for SNPs (Dallas, 1992; Ellegren, 2004; Lynch, 2010; Weber & Wong, 1993; Zhang & Hewitt, 2003). Combined with the larger number of possible alleles for a single locus, microsatellites provide immense levels of polymorphism, yielding high statistical power in population genetic inference (Allendorf, 2017; Avise, 2004). Microsatellites are very sensitive to sudden, or recent, demographical processes and are well suited to detect subtle population structure or recent bottlenecks (Haasl & Payseur, 2011; Luikart & Cornuet, 1998; Pereira, Teixeira, & Velo‐Antón, 2018; Putman & Carbone, 2014). However, high polymorphism is usually associated with homoplasy (Garza & Freimer, 1996; Hedrick, 1999; Queney, Ferrand, Weiss, Mougel, & Monnerot, 2001) and poses difficulties in fitting adequate evolutionary models to heterogeneous mutation processes (Ellegren, 2004; Di Rienzo et al., 1994; Valdes, Slatkin, & Freimer, 1993; Weber & Wong, 1993; Webster, Smith, & Ellegren, 2002). This can lead to unreliable estimates of divergence times (Kalinowski, 2002; Queney et al., 2001) and underestimation of genetic differentiation between populations caused by high intrapopulational heterozygosity (Hedrick, 1999). Furthermore, microsatellites are not well suited to reconstruct the evolutionary history of lineages or species under certain demographic scenarios, for instance, during range expansions, when consecutive founder events and allele surfing processes in newly formed populations inflate genetic differentiation (Pereira et al., 2018). A microsatellite locus contains from four to twelve times more information than a SNP (Liu, Chen, Wang, Oh, & Zhao, 2005). However, current genotyping costs for SNPs are relatively low, so the lower per‐locus information of SNPs is largely compensated by the sequencing of thousands of them at a similar cost than the genotyping of a few microsatellites (Hodel et al., 2016; Puckett, 2017). A large number of SNPs and their genome‐wide distribution secure a range of mutation rates that can, in principle, provide sufficient information at different evolutionary scales, from recent demographic processes within‐species to interspecies phylogenies (DeFaveri, Viitaniemi, Leder, & Merilä, 2013; Petersen et al., 2013).

The different molecular nature of SNPs and microsatellites is expected to impact their resolution power at different evolutionary scales, with microsatellites better reflecting recent demographic processes but rapidly losing resolution above the species level, and SNPs providing less information per locus but securing resolution of demographic processes over a wider evolutionary window (DeFaveri et al., 2013; Estoup, Jarne, & Cornuet, 2002; Haasl & Payseur, 2011). A review of the recent literature shows that thousands of SNPs are generally more powerful in detecting genetic structure than typical microsatellite datasets (Elbers, Clostio, & Taylor, 2017; Hodel et al., 2017; Jeffries et al., 2016; Malenfant, Coltman, & Davis, 2015; McCartney‐Melstad, Vu, & Shaffer, 2018; Puckett, 2017; Puckett & Eggert, 2016; Rašić, Filipović, Weeks, & Hoffmann, 2014). The choice of marker (SNPs versus microsatellites) also seems to affect estimates of the proportions of individual ancestries and the inferred optimal number of clusters (Bohling, Small, Von Bargen, Louden, & DeHaan, 2019; Bradbury et al., 2015; Elbers et al., 2017; Malenfant et al., 2015). These studies have made important contributions to our understanding of differences in patterns of genetic diversity and structure using both types of markers. However, the lack of comparable datasets, differences in the clustering methods used, and the absence of metrics allowing direct comparisons across marker types limit generalization of these results.

We present an explicit comparison of patterns of genetic structure and diversity based on comparable datasets of microsatellites and SNPs in two amphibian species: the Iberian tree frog, *Hyla molleri* Bedriaga, 1889, and the Western Spadefoot, *Pelobates cultripes* (Cuvier, 1829). Both are nearly endemic to the Iberian Peninsula (with some populations reaching southern France), and their range‐wide phylogeography has been previously investigated based on mitochondrial and microsatellite datasets (Gutiérrez‐Rodríguez, Barbosa, & Martínez‐Solano, 2017; Sánchez‐Montes, Recuero, Barbosa, & Martínez‐Solano, 2019). These studies linked their contrasting phylogeographic patterns with different demographic histories during the Late Quaternary. *Hyla molleri* is present in Continental and Atlantic Iberia, and its higher tolerance to colder conditions was hypothesized to account for their inferred demographic stability since the Last‐Glacial Maximum (~21,000 years ago; Sánchez‐Montes et al., 2019). In contrast, *P. cultripes* is a more thermophilous species present in southern and central Iberia, in areas with a Mediterranean influence. This species seems to have experienced important range contractions to southern glacial refugia during colder times in the Pleistocene, resulting in a south‐to‐north gradient of decreasing genetic diversity (Gutiérrez‐Rodríguez et al., 2017). The availability of comprehensive microsatellite datasets and the contrasting demographic histories in a shared geographical area make these two species good study systems for a robust comparative assessment of patterns of genetic diversity and structure obtained with microsatellites and SNPs.

## 2 MATERIALS AND METHODS

We used published microsatellite datasets for *H. molleri* and *P. cultripes* (Gutiérrez‐Rodríguez et al., 2017; Sánchez‐Montes et al., 2019) and generated SNP datasets for both species. Patterns of genetic structure between markers were compared based on model‐based clustering analyses and those of genetic diversity were assessed with individual heterozygosity estimates.

### 2.1 Data collection

Samples from *H. molleri* and *P. cultripes* covered most of their current ranges (Figure S1; Table S1). They were evenly distributed across the main genetic clusters determined in previous works with microsatellites (Gutiérrez‐Rodríguez et al., 2017; Sánchez‐Montes et al., 2019), securing the representation of more than 20 samples per north/south clusters. Microsatellite genotypes from *H. molleri* included 84 individuals from 25 localities genotyped at 18 loci (10% missing data) from Sánchez‐Montes, Recuero, Barbosa, and Martínez‐Solano (2019). Microsatellite genotypes from *P. cultripes* included 83 individuals from 43 localities genotyped at 14 loci (0% missing data) from Gutiérrez‐Rodríguez et al. (2017). To facilitate comparisons between marker datasets, we selected the same 83 individuals of *P. cultripes* for SNP genotyping. However, in *H. molleri* only 39 individuals from the microsatellite dataset were amenable for SNP genotyping. In this case, we sampled additional individuals from the same or nearby locations as represented in the original microsatellite study to complete a dataset of 90 individuals from 25 localities (Table S1; Figure S1).

Genomic DNA was extracted with ExtractMe Genomic DNA 96‐Well kits (DNA GDAŃSK), and concentrated with QIAamp DNA Micro (QIAGEN GmbtH) kits, when necessary. DNA extracts from *H. molleri* and *P. cultripes* were standardized to 500 ng of DNA (with exceptions as low as 390 ng) and sent for sequencing at Diversity Arrays Technology (Australia), which uses a proprietary protocol to sequence reduced representation of the genome from double‐digested restriction fragments. We chose DArTseq because it has been reported to work well with large and complex genomes, like those of amphibians (Lambert, Skelly, & Ezaz, 2016). The restriction fragments generated were sequenced in an Illumina HiSeq 2,500 as single‐end reads of 77 nucleotides (nt). The sequencing depths for *H. molleri* and *P. cultripes* were 7.7 and 5 million reads per sample, respectively. Diversity Arrays Technology provides genotypes from the proprietary DArTSoft14 pipeline in a text file along with several quality parameters on each SNP. Around 30% of the samples in the run are included as internal replicates to provide confidence levels on the genotype calls.

### 2.2 Data filtering

We applied several filtering steps to the SNP genotype matrices using R 3.6.0 (R Core Team, 2019) functions from the *dartR* 1.1.11 package (Gruber, Unmack, Berry, & Georges, 2018) and custom code. The filters were applied as follows. First, we retained samples with a proportion of loci with calls (call rate per individual) >0.35 and loci with high confidence on their genotype calls (*RepAvg* parameter from DArTseq >0.95). We kept loci with balanced alleles (proportion of reads for each allele across samples between 0.15 and 0.85) and removed loci whose coverage was 3.5 times higher than the median coverage across loci to remove potential paralogs (O’Leary, Puritz, Willis, Hollenbeck, & Portnoy, 2018). Then, we removed loci with a call rate (proportion of samples with a call) lower than 0.8, retained only one SNP per contig (the one with greatest repeatability) and removed alleles with a frequency <0.02 (O’Leary et al., 2018; Figure S2).

### 2.3 Genetic structure

We conducted model‐based genetic structure analyses in STRUCTURE v2.3.4 (Pritchard, Stephens, & Donnelly, 2000). For each dataset, we performed 10 replicate runs assuming a number of clusters (*K*) between 1 and 8 (*K *= 1 to *K* = 8), to encompass the optimal number of clusters (*K* = 2, *K* = 4, and *K* = 6) found in previous studies with microsatellites for the study species (Gutiérrez‐Rodríguez et al., 2017; Sánchez‐Montes et al., 2019), and explore any potential finer substructure. We used an admixture model with correlated allele frequencies (Falush, Stephens, & Pritchard, 2003) with no prior information on sample origin. For the microsatellite data, we used the same run lengths as in the original publications: 500,000 burnin steps followed by 1,000,000 iterations. For the SNP datasets, run lengths were shorter as data chains often converge faster: 30,000 burnin steps followed by 10,000 iterations (Table S2; Figures S3.1 and S3.2). For the SNP runs, we estimated lambda with *K* = 1 by averaging lambda estimates across three replicate runs. These values of lambda (0.67 for *H. molleri* and 0.69 for *P. cultripes*) were then used across all runs of the SNP data, whereas for microsatellites lambda was fixed to 1. Lower values of lambda can improve the modeling of correlated allele frequencies when using SNPs, where often the data are skewed toward rare alleles (Falush et al., 2003). We ran STRUCTURE in parallel in 8 cores using *Structure_threader* (Pina‐Martins, Silva, Fino, & Paulo, 2017), recording steps to log files every 50 and 5,000 iterations for the SNP and microsatellite data, respectively.

Convergence between the 10 replicate runs for each K was evaluated using Gelman and Rubin's convergence diagnostic, GR (Gelman & Rubin, 1992), with function *coda*::*gelman.diag* (Plummer, Best, Cowles, & Vines, 2006). Values below 1.05 indicate good convergence (Vats & Knudson, 2018). We used KFinder (Wang, 2019) to compare the best number of clusters for each dataset through three approaches: (a) Pr[X|K], the probability of data X given K clusters (Pritchard et al., 2000), (b) Evanno's ΔK, which considers the rate of change in the logarithm of the probability of data between successive K values (Evanno, Regnaut, & Goudet, 2005), and (c) PI, parsimony index, a newly proposed metric that favors K values yielding clusters with the most consistent and with minimal average individual admixture. The latter is assumed to be a more consistent metric across a wider range of demographic scenarios (Wang, 2019). We ran CLUMPAK (Kopelman, Mayzel, Jakobsson, Rosenberg, & Mayrose, 2015) on STRUCTURE outputs. CLUMPAK feeds the software CLUMPP with results of replicate runs for each K value to generate consensus solutions for the distinct modes. It also computes the similarity between Q‐matrices (ancestry matrices) from each run and matches clusters across successive values of K.

STRUCTURE results were contrasted with a model‐free hierarchical clustering method using the Neighbor‐Joining algorithm on pairwise genetic distances (File S1).

### 2.4 Congruence in ancestries between microsatellite and SNP datasets

We assessed the congruence of the Q‐matrices from STRUCTURE results between SNP and microsatellite datasets using the Symmetric Similarity Coefficient (SSC; Jakobsson & Rosenberg, 2007). For *P. cultripes*, since all individuals were identical among datasets, we ran CLUMPAK over the combined STRUCTURE results from both markers (*n* = 20 runs per K). The CLUMPP algorithm in CLUMPAK computes a pairwise distance matrix for all runs in each K based on the SSC. For *H. molleri*, since we sampled different individuals from the same localities for microsatellites and SNPs, we averaged individual ancestries per locality and used R package *starmie* 0.1.2 (Tonkin‐Hill & Lee, 2016) to run CLUMPP and compute the similarity coefficients. SSC ranges from negative values to a maximum of 1 when Q‐matrices are identical. Pairwise SSCs were computed between runs from the same marker (SNPs‐SNPs, microsatellites‐microsatellites), in addition to cross‐comparisons between markers (SNPs‐microsatellites). To aid visualization of spatial patterns of genetic structure, we computed mean ancestries per locality for each species and marker from major clusters after CLUMPAK results. Then, for each species and *K* value, we aligned the microsatellite and SNP matrices using the CLUMPP algorithm from *starmie* 0.1.2.

We evaluated admixture in individual ancestries of *P. cultripes* for each K in STRUCTURE using a newly developed index: the Coefficient of Admixture, CA. CA*_Ki_* for individual *i* across clusters of a Q‐matrix from a given *K* in STRUCTURE represent individual levels of genetic admixture, 0 indicating all ancestry belonging to a single cluster, and 1, equal proportions across clusters (details in File S2).

### 2.5 Genetic diversity

Individual heterozygosity with each marker type was computed as the proportion of heterozygous loci standardized by the heterozygosity of loci across the dataset (standardized multilocus heterozygosity, sMLH; Coltman, Pilkington, Smith, & Pemberton, 1999), using *inbreedR::sMLH* (Stoffel et al., 2016) in R. We then represented the median sMLH per locality for each dataset in a map to describe the spatial distribution of genetic diversity. Pearson correlations were computed between sMLH from microsatellite and SNP data for individuals of *P. cultripes*. We also explored patterns of genetic diversity along the axes of demographic expansions from inferred glacial refugia in both species. For that purpose, the putative effects of latitudinal and longitudinal gradients on patterns of genetic diversity were assessed with linear models in R, using sMLH as a dependent variable and latitude and longitude as fixed effects.

## 3 RESULTS

We produced panels of 15,412 SNPs (7.6% missing data) for 90 individuals of *H. molleri* and 33,140 SNPs (5.2% missing data) for 83 individuals of *P. cultripes*.

### 3.1 Genetic structure

STRUCTURE runs converged well for low K values but not for larger K values (Table S2; Figures S3.1 and S3.2). The best‐supported number of genetic clusters (K) identified using STRUCTURE varied according to the metric used (PI or ΔK) and marker type. In most cases, we found the best support for two genetic lineages (*K* = 2), but some metrics identified further substructure, with up to six genetic clusters (*K* = 6) when using PI (Table S3; Figures S4.1 and S4.2).

**Figure 1:**
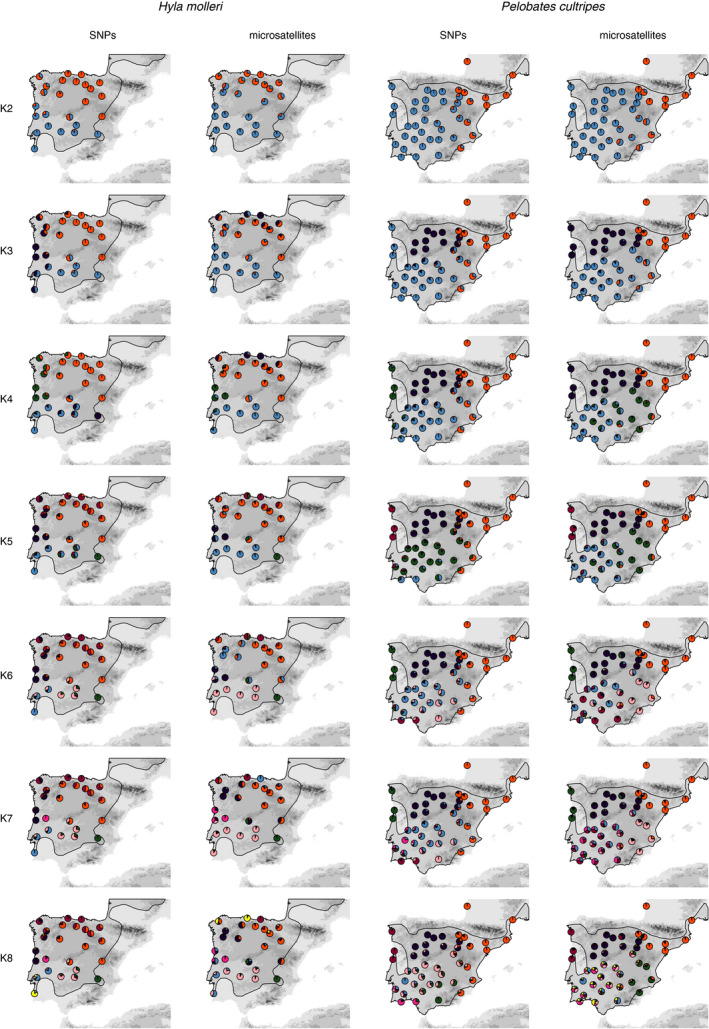
Genetic structure in *Hyla molleri* (left) and *Pelobates cultripes* (right) based on STRUCTURE analyses of the SNP and microsatellite datasets. Pies represent averaged proportion of inferred ancestries of the major mode in CLUMPAK, from *K *= 2 to *K *= 8. Shaded areas represent the species distributions. To facilitate visual comparison of spatial patterns of genetic structure between markers, Q‐matrices from both markers for any given K and species were aligned using CLUMPP before plotting

Ancestries derived from both markers were spatially coherent at different K values. That is, individuals from the same or nearby localities shared similar ancestries and more admixed individuals coincided with geographical shifts in cluster assignment (Figure 1). For *K* = 2, both marker types were congruent in identifying major subdivisions in each species: a northern and a southern lineage for *H. molleri*, and a central‐western and a northeastern lineage for *P. cultripes*. From *K* = 3 to *K* = 8, the spatial patterns of genetic structure for both species were largely congruent between marker types in terms of admixture levels and ancestry group assignment (Figure 1 and Figure S5). Both markers generally agreed on the genetic ancestry of localities or group of localities as sharing a singular genetic ancestry, although the K value at which for a given assignment to a cluster could differ between markers. For instance, for *H. molleri*, the western‐coastal populations from Portugal (dark purple, Figure 1) formed a well‐differentiated cluster at *K* = 3 with SNPs and at *K* = 4 with microsatellites. Another example is the locality Ojos de Villaverde, at the southeastern‐most corner of the distribution of *H. molleri*. This locality appeared well differentiated at *K* = 4 for SNPs (green), but at *K* = 5 in microsatellites (magenta) (Figure 1). In *P. cultripes*, we observed the same phenomenon. For instance, the localities from northwestern Portugal were very differentiated at *K* = 4 with SNPs (green), but at *K* = 5 with microsatellites (green, Figure 1). Both markers agreed in individuals from localities within the northern half of the Iberian Peninsula with nearly “pure” ancestries and no further clustering after *K* = 4, and yielded more admixed individuals in the southern half of Iberia from *K* = 4 to *K* = 8, although the levels of admixture and the ancestry assignments differed notably between markers. In *P. cultripes*, for *K* = 7 and *K* = 8, microsatellites yielded more admixed individual ancestries compared with SNPs (Figure S5), driven by the more admixed southern populations (Figure 1). For *H. molleri*, we could not quantify reliably these differences in admixture levels between markers because the individuals analyzed for each dataset were not all the same.

Genetic structure based on STRUCTURE analyses was highly congruent with that inferred by model‐free hierarchical clustering (File S1), which yielded well‐supported clades for SNPs but less so in microsatellite‐based topologies.

### 3.2 Congruence in individual/locality ancestries between microsatellites and SNPs

**Figure 2:**
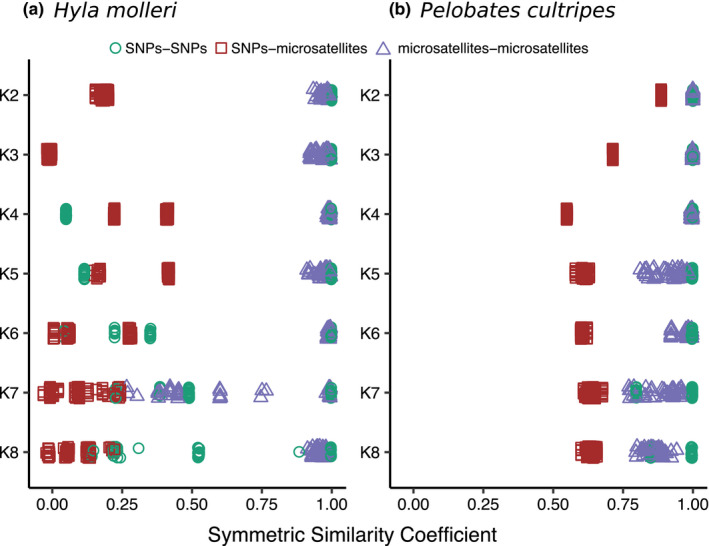
Comparison of STRUCTURE results in the SNP and microsatellite datasets for *H. molleri* (a) and *P. cultripes* (b). The horizontal axis shows Pairwise Symmetric Similarity Coefficients between Q‐matrices from STRUCTURE runs across K values (vertical axis) using averaged ancestries per locality in *H. molleri* and individual ancestries in *P. cultripes*. Comparisons involving the same marker type (microsatellite‐microsatellite: blue triangles, and SNP‐SNP: green circles) show higher similarity than those involving different marker types (red squares)

Both species showed higher intramarker similarity (*H. molleri*, SSCs = 0.27–1.00; *P. cultripes*, SSCs = 0.77–1.00) than intermarker similarity (*H. molleri*, SSCs = −0.03 to – 0.42; *P. cultripes*, SSCs = 0.55–0.89) (Figure 2). For microsatellites, ancestries were very similar (SSCs close to 1) from *K* = 2 to *K* = 8 (except *K* = 7) for *H. molleri* and from *K* = 2 to *K* = 4 for *P. cultripes*. For SNPs, STRUCTURE results were almost identical only from *K* = 2 to *K* = 4 for *H. molleri*, but up to *K* = 6 for *P. cultripes*. Larger *K* values were in all cases associated with less consistent results across STRUCTURE runs. For most *K* values, pairwise SSC values in microsatellite runs had a larger spread (i.e., a greater range of values), especially at larger *K* values. This spread was minimum for STRUCTURE results derived from SNPs, though at larger *K* values (*K* = 4 to *K* = 8 for *H. molleri*; *K* = 6 to *K* = 8 for *P. cultripes*) they tended to converge into 2 or even 3 regions of the parameter space (Figure 2). The similarity between SNP‐microsatellite runs did not follow a clear pattern along increasing *K*. For *H. molleri*, SSCs were homogenously lower across all *K* values than for *P. cultripes*, highlighting the distinct solutions obtained between datasets. For this species, SSCs were maximum at *K *= 2 (0.89) and minimum at *K *= 4 (0.55). From *K *= 5 to *K *= 8, SSCs had a small increase in the 0.58–0.68 range.

Microsatellites yielded more admixed ancestries at larger values of *K* (i.e., *K *= 7 and *K *= 8; Figure S5) which seem to be driven by the more complex patterns of genetic structure in the southern localities (Figure 1).

### 3.3 Genetic diversity

**Figure 3:**
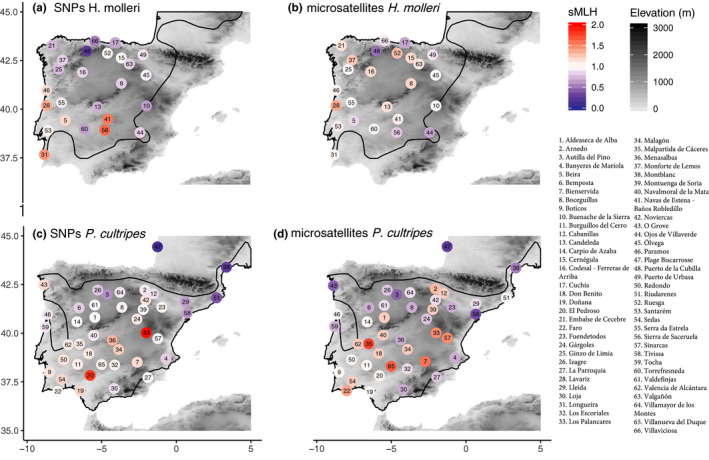
Genetic diversity measured as multilocus heterozygosity (sMLH) for *H. molleri* (a: SNPs, b: microsatellies) and *P. cultripes* (c: SNPs, d: microsatellites)

Correlation of genetic diversity between microsatellites and SNPs‐based measures was significant but weak (*P. cultripes*, Pearson's *r* = 0.39, *p* < .001). Genetic diversity (sMLH) from SNPs in *H. molleri* was highest in southwest Iberia and decreased toward northern (*β* = −0.08; *p* < .001) and eastern localities (*β* = −0.04; *p* = .02) (Figure 3; Table S4). We did not detect a significant correlation of microsatellite diversity with latitude (*p* = .63) or longitude (*p* = .10).

For *P. cultripes*, genetic diversity decreased with latitude for SNPs (*β* = −0.07; *p* < .001) and microsatellites (*β* = −0.09; *p* < .001). Longitude had a marginal effect on diversity from SNPs (*β* = −0.02; *p* = .06) but not from microsatellites (*p* = .93). Both markers agreed in diversity being (a) extremely low in the northeastern localities, in coastal France, both on the Atlantic and Mediterranean sides, (b) moderately low in the Northern Plateau and along the Mediterranean coast and interior, and (c) greatest in the central southwestern localities (Figure 3; Table S4). These southwestern localities also showed the largest complexity in genetic structure and patterns of admixture across K (Figure 1).

## 4 DISCUSSION

Our comparative assessment revealed that a typical microsatellite dataset (18 loci in *H. molleri* and 14 in *P. cultripes*) can yield similar range‐wide patterns of genetic structure than those inferred with a few thousand SNPs (15,412 and 33,140, respectively). Differences across marker types involved mainly inference of the optimal number of clusters (*K*), and assessment of individual and population admixture levels.

### 4.1 Effect of marker type in model‐based clustering and genetic diversity

We found overall concordance between markers in recovering the same major genetic clusters in STRUCTURE analyses (Figure 1), although the model‐free clustering approach based on NJ yielded poorly supported clustering for microsatellites compared with SNPs (File S1). This shows that population genetic models implemented in STRUCTURE are efficient to infer genetic structure from a few highly polymorphic microsatellite loci, with comparable performance to analyses using thousands of bialellic SNPs. Many population genetics studies often rely on a few microsatellites compared with the hundreds or thousands of SNPs needed to address similar questions regarding population structure (Haasl & Payseur, 2011; Puckett, 2017). Previous research comparing both marker types claimed that SNPs offered a “better” resolution to address biological questions when compared to microsatellites, usually referring to SNPs being able to identify more differentiated genetic clusters (Elbers et al., 2017; Hodel et al., 2017; Jeffries et al., 2016; Malenfant et al., 2015; McCartney‐Melstad et al., 2018; Puckett & Eggert, 2016; Rašić et al., 2014). These assertions in favor of SNPs over microsatellites could potentially be exaggerated, because they mostly derive from nonparametric (e.g., PCA or DAPC) instead of model‐based methods.

There were, however, discordances between markers. The inferred optimal number of clusters was not consistent across marker types and method of estimation (Figure S4; Table S3). The clear peak of Δ*K* at *K *= 2 in the SNP dataset in *H. molleri* contrasted with the peak of Δ*K* at *K *= 4 and *K *= 6 in microsatellites in our results and in Sánchez‐Montes et al. (2019), respectively. Also, the PI pointed to higher larger optimal values of K than those selected by Evanno's Δ*K* (Figure S4). The clear peaks of Δ*K* at *K *= 2 in the SNP datasets describe the top level of hierarchical population structure and must be interpreted cautiously, since *K *= 2 is the optimal *K* value most often reported across studies even when further genetic substructure is present (Janes et al., 2017). The number of samples per population can have a strong effect on the optimal *K* value inferred (Puechmaille, 2016). Furthermore, the history of populations is often more complex than the “top‐level” clustering approach in STRUCTURE, and as *K* increases, violations in the assumptions of STRUCTURE may hamper the inference of the correct population structure (Lawson, van Dorp, & Falush, 2018).

Inferred ancestral groups (clusters) and their proportions of ancestry were not fully congruent between marker types. The similarity in the Q‐matrices between markers varied for both species across *K*. This was evidenced by ancestral groups arising at different *K* depending on the marker type and the different characterization of ancestral groups reflected in the amount of admixture and spatial extent of the clusters (Figure 1). Also, genetic admixture was higher in microsatellites than in SNPs only at larger *K* values for *P. cultripes*, driven by the localities with higher genetic diversity (central and southern Iberia; Figure 1 and Figure S5). Greater genetic admixture detected by microsatellites, together with their greater variance in STRUCTURE solutions at large values of *K* (Figure 2), suggest microsatellites have reduced power to detect weaker or more complex signals of genetic structure, as those reflected at larger values of *K*. For SNPs, even at larger values of *K* (*K *> 6), the SSC fell into alternative discrete solutions (Figure 2). These alternative solutions to the optimal *K* problem deserve independent biological interpretations (Kopelman et al., 2015; Pritchard et al., 2000; Wang et al., 2007), but should be considered with caution to avoid over‐interpretation (Lawson et al., 2018). Previous studies comparing STRUCTURE results between SNPs and microsatellites used datasets or approaches that were not fully comparable between the two marker types, limiting the scope of their conclusions. For instance, Bradbury et al. (2015) described different levels of admixture between markers but used different biological samples for each marker type, while Bohling et al., (2019) relied on different clustering approaches, NGSadmix (for SNP data) and STRUCTURE (for microsatellites), to conclude that microsatellites yielded less precise and less consistent results. Of the few studies that used exactly the same individuals and clustering approach across different marker types, Lemopoulos et al. (2019) found nearly identical ancestry memberships, whereas Malenfant et al. (2015) reported more admixed ancestries for microsatellites, in agreement with our results.

Genetic diversity decreased with latitude in SNPs for both species but only in *P. cultripes* for microsatellites. Genetic diversity as estimated from SNPs was spatially more coherent with genetic structure, showing less variance between localities from the same cluster (e.g., the southern group of *P. cultripes*; Figure 3). The differences in genetic diversity between marker types resulted in a weak correlation of the corresponding sMLH values (Figure S6). The high number of SNPs may overcome some of the limitations of using few loci as surrogates of genome‐wide variation, like stochasticity related to loci selection and the associated ascertainment bias (Fischer et al., 2017; Guillot & Foll, 2009; Lemopoulos et al., 2019; Morin et al., 2004). Different marker discovery approaches (e.g., representation of functional genomic regions) could be related to some of the differences between markers (Clark, Hubisz, Bustamante, Williamson, & Nielsen, 2005; Dufresnes, Brelsford, Béziers, & Perrin, 2014; Lachance and Tishkoff, 2013). Additionally, differences in type and rate mutation could also account for the differences in patterns between markers (Ellegren, 2004; Morin et al., 2004). The better representation of loci covering a wider evolutionary scale in SNPs (Haasl & Payseur, 2011; Linck & Battey, 2019; Morin et al., 2004) could be responsible for some loss of resolution when using microsatellites to infer older demographic processes. This suggests that microsatellites might be offering suboptimal measures of genomic diversity (Fischer et al., 2017; Väli, Einarsson, Waits, & Ellegren, 2008).

### 4.2 Contribution of SNPs to unravelling the evolutionary history of *H. molleri* and *P. cultripes*

Our SNP results on *H. molleri* are consistent with those of Sánchez‐Montes et al. (2019) in recovering two major clusters, southern and northern, coinciding with the two major mitochondrial lineages and the north/south microsatellite clusters. Patterns of genetic diversity as measured with SNPs decrease with latitude and decrease from coastal localities of central Portugal toward the east (Figure 3). Sánchez‐Montes et al. (2019) also found greater mitochondrial and microsatellite diversity in western localities, but no clear association with latitude. Our findings support the existence of southwestern refugia for *H. molleri* in Iberia, where it would have persisted through glacial cycles in Atlantic central‐south Portugal and Sierra Morena, followed by two major historical dispersal axes, toward the north and east.

For *P. cultripes*, analysis of the SNP dataset provided results congruent with microsatellites and mitochondrial DNA from Gutiérrez‐Rodríguez et al. (2017), identifying three main lineages: a southern one with high genetic diversity and complex genetic structure, a second lineage in the Northern Plateau with low genetic diversity, and a third lineage in the northeast, with very low genetic diversity (Figures 1 and 3). The two latter groups were not further substructured, but we found signs of finer substructure in the southern lineage. Our results support northern and eastern colonization routes from southern refugia, with the Northern‐Plateau lineage probably resulting from a relatively recent colonization event, contrasting with the interpretation of Gutiérrez‐Rodríguez et al. (2017), who suggested the existence of a Northern‐Plateau refugium. This study thus adds to the growing body of evidence showing the importance of southern refugia for a broad range of taxa in the Iberian Peninsula across glaciations in the Pleistocene (Gómez & Lunt, 2007). Inferred trends of decreasing genomic diversity toward northern latitudes provide valuable information for the management of the genetically diverse populations from southern refugia and their less diverse northern counterparts, both of which face increased risk of extinction under future climatic scenarios (Araújo, Guilhaumon, Neto, Ortego, & Calmaestra, 2011).

## CONFLICT OF INTEREST

The authors declare that they have no conflict of interest.

## AUTHOR CONTRIBUTION

**Miguel Camacho‐Sanchez:** Conceptualization (lead); Data curation (lead); Formal analysis (lead); Methodology (lead); Visualization (lead); Writing‐original draft (lead). **Guillermo Velo‐Antón:** Conceptualization (lead); Formal analysis (supporting); Methodology (supporting); Resources (lead); Supervision (lead); Writing‐review & editing (supporting). **Jeffrey O Hanson:** Data curation (supporting); Formal analysis (supporting); Methodology (supporting); Writing‐review & editing (supporting). **Ana Verissimo:** Conceptualization (equal); Formal analysis (supporting); Project administration (supporting); Supervision (supporting); Writing‐review & editing (supporting). Í**ñigo Martínez‐Solano:** Conceptualization (equal); Methodology (supporting); Resources (lead); Supervision (supporting); Writing‐review & editing (supporting). **Adam D. Marques:** Data curation (supporting); Methodology (supporting); Project administration (supporting); Resources (supporting); Writing‐review & editing (supporting). **Craig Moritz:** Conceptualization (supporting); Formal analysis (supporting); Project administration (supporting); Writing‐review & editing (supporting). **Sílvia Carvalho:** Conceptualization (equal); Funding acquisition (lead); Project administration (lead); Supervision (equal); Writing‐review & editing (supporting).

### OPEN RESEARCH BADGES

This article has earned an Open Data Badge for making publicly available the digitally‐shareable data necessary to reproduce the reported results. The data is available at: 10.5281/zenodo.3953536 and https://github.com/csmiguel/usat_snp.

## Supporting information

Supplementary Material

## Data Availability

Raw data, genotypes, code for data analysis, and the generation of figures are available in a GitHub repository (github.com/csmiguel/usat_snp), and in a permanent release deposited in ZENODO (https://doi.org/10.5281/zenodo.3953536).
