# Supplementary material for: Comparative assessment of range‐wide patterns of genetic diversity and structure with SNPs and microsatellites: A case study with Iberian amphibians
Source: Ecol Evol. 2020 Sep 15;10(19):10353–63. doi: 10.1002/ece3.6670 (PMC7548196; doi:10.1002/ece3.6670)
Supplement: Supplementary file 1 — Supplementary Material [file ECE3-10-10353-s001.docx]

**Supplemental Information for:**

**Comparative assessment of range-wide patterns of genetic diversity and structure with SNPs and microsatellites: a case study with Iberian amphibians**

Miguel Camacho-Sanchez, Guillermo Velo-Antón, Jeffrey O. Hanson, Ana Veríssimo, Íñigo Martínez-Solano, Adam Marques, Craig Moritz & Sílvia B. Carvalho

**Table of Contents:**

Supplementary Tables:

[Table S1. Samples of](#_Toc43396954) *[H. molleri](#_Toc43396954)* [and](#_Toc43396954) *[P. cultripes](#_Toc43396954)* [used in data analysis after filtering steps, including locality information (latitude, longitude) and sample codes. 2](#_Toc43396954)

[Table S2. Gelman and Rubin's convergence diagnostic values for STRUCTURE runs of full and subsampled datasets of SNPs (A) and microsatellites (B). 5](#_Toc43396955)

[Table S3. Variation in metrics evaluating the optimal K values across marker types for K = 2 to K = 8. The optimal K is shown in bold font in each case. 6](#_Toc43396956)

[Table S4. Genetic diversity (sMLH) per individual and median per locality for both marker types. 7](#_Toc43396957)

[Figure S1. Sample localities. 12](#_Toc43396958)

[Figure S2. DArTseq genotype filtering. 13](#_Toc43396959)

[Figure S3. Likelihood of the STRUCTURE runs. 14](#_Toc43396960)

[Figure S4. Most optimal K. 15](#_Toc43396961)

[Figure S5. Admixture for STRUCTURE results. 16](#_Toc43396962)

[Figure S6. Residuals from genetic diversity. 17](#_Toc43396963)

[Supplementary File S1. Model free hierarchical clustering. 18](#_Toc43396964)

[Supplementary File S2. Coefficient of admixture 22](#_Toc43396965)

## Table S1. Samples of *H. molleri* and *P. cultripes* used in data analysis after filtering steps, including locality information (latitude, longitude) and sample codes.

| ID | locality | longitude | latitude | *H. molleri* | *P. cultripes* |
| --- | --- | --- | --- | --- | --- |
| 1 | Aldeaseca de Alba | 40.813 | -5.454 | - | PC605-6 |
| 2 | Arnedo | 42.257 | -2.069 | - | IMS3888, IMS3890 |
| 3 | Autilla del Pino | 41.990 | -4.628 | - | IMS3062, IMS3067 |
| 4 | Banyeres de Mariola | 38.727 | -0.712 | - | IMS3163 |
| 5 | Beira | 39.428 | -7.391 | SNPs: GVA7392-6  microsatellites: MNCN8932 |  |
| 6 | Bemposta | 41.317 | -6.498 | - | IMS2814 |
| 7 | Bienservida | 38.533 | -2.662 | - | IMS3226-7, IMS3229 |
| 8 | Boceguillas | 41.331 | -3.622 | SNPs: IMS4064-8  microsatellites: IMS4065-7 | IMS4074-5 |
| 9 | Boticos | 38.005 | -8.496 | - | PC470 |
| 10 | Buenache de la Sierra | 40.179 | -1.943 | SNPs: IMS4158, IMS4163-6  microsatellites: IMS4157-9 | - |
| 11 | Burguillos del Cerro | 38.381 | -6.620 | - | PC557 |
| 12 | Cabanillas | 42.046 | -1.506 |  | IMS3898 |
| 13 | Candeleda | 40.126 | -5.221 | SNPs: HaT3  microsatellites: HaT3 | - |
| 14 | Carpio de Azaba | 40.594 | -6.628 |  | PC659-60 |
| 15 | Cernégula | 42.639 | -3.624 | SNPs: HaMj1  microsatellites: HaMj1 | - |
| 16 | Codesal - Ferreras de Arriba | 41.929 | -6.288 | SNPs: IMS4205, IMS4207  microsatellites: IMS4214-6, IMS4264-5, IMS4267 | - |
| 17 | Cuchía | 43.431 | -4.029 | SNPs: IMS4690-4  microsatellites: IMS4690-8 | - |
| 18 | Don Benito | 38.958 | -5.854 | - | PC585 |
| 19 | Doñana | 37.016 | -6.444 | - | PC700-2 |
| 20 | El Pedroso | 37.835 | -5.776 | - | PC406, PC408 |
| 21 | Embalse de Cecebre | 43.275 | -8.282 | SNPs: HaGa2  microsatellites: HaGa1 | - |
| 22 | Faro | 37.035 | -7.955 | - | PC757-9 |
| 23 | Fuendetodos | 41.340 | -0.946 | - | IMS2294 |
| 24 | Gárgoles | 40.736 | -2.626 | - | IMS2616, IMS2626 |
| 25 | Ginzo de Limia | 42.054 | -7.821 | SNPs: IMS3550-1, IMS3554  microsatellites: IMS3549-51 | - |
| 26 | Izagre | 42.221 | -5.263 | - | IMS3173-4 |
| 27 | La Parroquia | 37.739 | -1.919 | - | IMS4385-6 |
| 28 | Lavariz | 40.196 | -8.642 | SNPs: IMS1448-52  microsatellites: IMS1448-50 | - |
| 29 | Lleida | 41.571 | 0.648 | - | PC846, PC848 |
| 30 | Loja | 37.140 | -4.161 | - | PC941-3 |
| 31 | Longueira | 37.672 | -8.777 | SNPs: IMS4177, IMS4180, IMS4182-4  microsatellites: IMS4177-9, IMS4185* | - |
| 32 | Los Escoriales | 38.276 | -4.024 | - | PC377 |
| 33 | Los Palancares | 40.028 | -1.995 | - | IMS3349, IMS3351 |
| 34 | Malagón | 39.173 | -3.882 | - | PC650-1 |
| 35 | Malpartida de Cáceres | 39.458 | -6.492 | - | PC780 |
| 36 | Menasalbas | 39.651 | -4.320 | - | PC628-30 |
| 37 | Monforte de Lemos | 42.529 | -7.575 | SNPs: IMS3536B  microsatellites: IMS3534B, IMS3535, IMS3537 | - |
| 38 | Montblanc | 43.378 | 3.361 | - | PC885 |
| 39 | Montuenga de Soria | 41.224 | -2.211 | - | IMS2612 |
| 40 | Navalmoral de la Mata | 39.901 | -5.540 | - | IMS2800-1 |
| 41 | Navas de Estena - Baños Robledillo | 39.496 | -4.518 | SNPs: IMS2166-7, IMS2170-1  microsatellites: IMS2166-8, IMS4498 | - |
| 42 | Noviercas | 41.712 | -2.031 | - | PC821-3 |
| 43 | O Grove | 42.473 | -8.870 | - | PC722-3 |
| 44 | Ojos de Villaverde | 38.806 | -2.369 | SNPs: IMS4823-6, IMS4828-9  microsatellites: IMS4828, IMS4830, IMS4839, IMS4842 | - |
| 45 | Ólvega | 41.752 | -1.962 | SNPs: IMS4100-3, IMS4105  microsatellites: IMS4097-9 | - |
| 46 | Paramos | 40.979 | -8.641 | SNPs: IMS3542-5  microsatellites: IMS3542-4 | PC679, PC681 |
| 47 | Plage Biscarrosse | 44.452 | -1.248 | - | IMS3980-1 |
| 48 | Puerto de la Cubilla | 42.990 | -5.926 | SNPs: IMS3238  microsatellites: IMS3235-7 | - |
| 49 | Puerto de Urbasa | 42.797 | -2.172 | SNPs: IMS3932-4  microsatellites: IMS3931-3 | - |
| 50 | Redondo | 38.650 | -7.598 | - | PC737-9 |
| 51 | Riudarenes | 41.828 | 2.716 | - | PC865-6 |
| 52 | Ruesga | 42.883 | -4.548 | SNPs: IMS4725-9  microsatellites: IMS4725-9 | - |
| 53 | Santarém | 38.992 | -8.528 | SNPs: ERG1275, GVA7411-4  microsatellites: ERG1275-6, ERG1285 | - |
| 54 | Sedas | 37.587 | -7.632 | - | PC450-1 |
| 55 | Serra da Estrela | 40.342 | -7.626 | SNPs: IMS3555-7, IMS3559A  microsatellites: IMS3555-7, IMS4235-7 | - |
| 56 | Sierra de Saceruela | 38.911 | -4.701 | SNPs: IMS4658-60, IMS4664, IMS4666-7  microsatellites: IMS3816 | - |
| 57 | Sinarcas | 39.748 | -1.234 | - | IMS3329-30 |
| 58 | Tivissa | 41.014 | 0.700 | - | PC926-7, PC930 |
| 59 | Tocha | 40.337 | -8.783 | - | PC800-1 |
| 60 | Torrefresneda | 38.992 | -6.096 | SNPs: IMS2925-6  microsatellites: IMS2922-4 | - |
| 61 | Valdefinjas | 41.444 | -5.460 | - | IMS3119-20 |
| 62 | Valencia de Alcántara | 39.428 | -7.234 | - | PC808-9 |
| 63 | Valgañón | 42.327 | -3.089 | SNPs: IMS3856-9  microsatellites: IMS3856-8 | - |
| 64 | Villamayor de los Montes | 42.100 | -3.750 | - | IMS4045 |
| 65 | Villanueva del Duque | 38.352 | -5.003 | - | PC394-5 |
| 66 | Villaviciosa | 43.518 | -5.384 | SNPs: IMS4703-4  microsatellites: IMS4701-4, IMS4854 |  |

## Table S2. Gelman and Rubin's convergence diagnostic values for STRUCTURE runs of full and subsampled datasets of SNPs (A) and microsatellites (B).

| A. SNPs | | | | | | | | | | | | | | | | | |
| --- | --- | --- | --- | --- | --- | --- | --- | --- | --- | --- | --- | --- | --- | --- | --- | --- | --- |
| Species | No. of loci | | | K1 | | K2 | | K3 | | K4 | | K5 | | | K6 | K7 | K8 |
| *H. molleri* | 200 | | | 1.00 | | 1.00 | | 1.00 | | 4.10 | | 7.13 | | | 2.05 | 10.66 | 3.18 |
|  | 500 | | | 1.00 | | 1.00 | | 5.16 | | 6.24 | | 10.42 | | | 11.72 | 16.43 | 13.78 |
|  | 1000 | | | 1.00 | | 1.00 | | 1.00 | | 7.77 | | 16.97 | | | 17.51 | 25.93 | 24.01 |
|  | 3000 | | | 1.00 | | 1.00 | | 1.00 | | 17.95 | | 28.24 | | | 34.88 | 29.07 | 9.99 |
|  | 5000 | | | 1.00 | | 1.00 | | 1.00 | | 17.02 | | 29.60 | | | 44.57 | 1.64 | 21.81 |
|  | 10000 | | | 1.00 | | 1.01 | | 1.06 | | 20.75 | | 40.38 | | | 43.25 | 2.88 | 81.56 |
|  | 15412 | | | 1.00 | | 1.00 | | 1.00 | | 23.07 | | 50.13 | | | 49.97 | 24.89 | 106.9 |
| *P. cultripes* | 200 | | | 1.00 | | 1.00 | | 1.00 | | 1.00 | | 1.26 | | | 1.21 | 1.35 | 2.18 |
|  | 500 | | | 1.00 | | 1.00 | | 1.00 | | 1.00 | | 2.48 | | | 1.00 | 1.07 | 1.60 |
|  | 1000 | | | 1.00 | | 1.00 | | 1.00 | | 1.04 | | 1.00 | | | 2.18 | 1.00 | 2.00 |
|  | 3000 | | | 1.00 | | 1.00 | | 1.00 | | 1.00 | | 1.01 | | | 1.01 | 1.83 | 6.51 |
|  | 5000 | | | 1.00 | | 1.00 | | 1.00 | | 1.00 | | 1.00 | | | 1.01 | 10.91 | 13.11 |
|  | 10000 | | | 1.00 | | 1.00 | | 1.00 | | 1.00 | | 1.00 | | | 18.91 | 21.02 | 17.72 |
|  | 20000 | | | 1.00 | | 1.00 | | 1.00 | | 1.00 | | 1.02 | | | 1.13 | 30.50 | 32.51 |
|  | 33140 | | | 1.00 | | 1.00 | | 1.00 | | 1.00 | | 1.01 | | | 1.01 | 39.16 | 33.34 |
| B. microsatellites | | | | | | | | | | | | | |  |  |  |  |
| Species | 1 | 2 | 3 | | 4 | | 5 | | 6 | | 7 | | 8 |  |  |  |  |
| *H. molleri* | 1.00 | 1.00 | 1.01 | | 1.00 | | 1.02 | | 1.00 | | 1.50 | | 1.05 |  |  |  |  |
| *P. cultripes* | 1.00 | 1.00 | 1.00 | | 1.00 | | 1.00 | | 1.00 | | 1.05 | | 1.03 |  |  |  |  |

## Table S3. Variation in metrics evaluating the optimal K values across marker types for K = 2 to K = 8. The optimal K is shown in bold font in each case.

| species | marker | metric | K1 | K2 | K3 | K4 | K5 | K6 | K7 | K8 |
| --- | --- | --- | --- | --- | --- | --- | --- | --- | --- | --- |
| *H. molleri* | microsatellites | Pr[X\|K] | -5429 | -5149 | -4956 | -4796 | -4884 | **-4597** | -4745 | -4636 |
|  |  | sd Pr[X\|K] | 0.71 | 4.37 | 2.70 | 4.10 | 61.53 | 4.57 | 351.58 | 62.95 |
|  |  | Evanno ΔK | - | 19.92 | 12.18 | 60.55 | 6.10 | **95.28** | 0.73 | - |
|  |  | Parsimony Index | 0.50 | 0.74 | 0.84 | **0.90** | 0.69 | 0.76 | 0.51 | 0.59 |
|  | SNPs | Pr[X\|K] | -987431 | -826356 | -768781 | -735661 | -705374 | -686670 | -670457 | -**667672** |
|  |  | sd Pr[X\|K] | 64.60 | 119.57 | 241.77 | 2814.14 | 7651.68 | 8649.81 | 4347.40 | 19694.38 |
|  |  | Evanno ΔK | - | **865.59** | 101.15 | 1.01 | 1.51 | 0.29 | 3.09 | - |
|  |  | Parsimony Index | 0.50 | 0.77 | 0.87 | 0.91 | **0.95** | 0.62 | 0.73 | 0.57 |
| *P. cultripes* | microsatellites | Pr[X\|K] | -2996 | -2609 | -2491 | -2429 | -2400 | **-2381** | -2396 | -2410 |
|  |  | sd Pr[X\|K] | 0.79 | 0.16 | 0.52 | 1.32 | 9.26 | 4.97 | 18.54 | 19.45 |
|  |  | Evanno ΔK | - | **1704.53** | 108.23 | 24.39 | 1.16 | 6.78 | 0.09 | - |
|  |  | Parsimony Index | 0.50 | 0.78 | 0.86 | 0.90 | 0.90 | **0.96** | 0.75 | 0.74 |
|  | SNPs | Pr[X\|K] | -2080297 | -1677497 | -1572564 | -1526066 | -1496957 | -1476625 | -1462820 | **-1453714** |
|  |  | sd Pr[X\|K] | 139.51 | 275.02 | 208.32 | 737.34 | 1645.70 | 1395.14 | 7076.54 | 5197.58 |
|  |  | Evanno ΔK | - | **1083.08** | 280.50 | 23.58 | 5.33 | 4.68 | 0.66 | - |
|  |  | Parsimony Index | 0.50 | 0.78 | 0.86 | **0.92** | 0.75 | 0.63 | 0.48 | 0.57 |

## Table S4. Genetic diversity (sMLH) per individual and median per locality for both marker types.

|  |  |  | **SNPs** | | **microsatellites** | |
| --- | --- | --- | --- | --- | --- | --- |
| **species** | **sample_id** | **locality** | **individual** | **median locality** | **individual** | **median locality** |
| *H. molleri* | GVA7394 | Beira | 1.18 | 1.18 | NA | 0.85 |
| *H. molleri* | GVA7395 | Beira | 1.05 | 1.18 | NA | 0.85 |
| *H. molleri* | GVA7396 | Beira | 1.63 | 1.18 | NA | 0.85 |
| *H. molleri* | GVA7392 | Beira | 1.49 | 1.18 | NA | 0.85 |
| *H. molleri* | GVA7393 | Beira | 0.69 | 1.18 | NA | 0.85 |
| *H. molleri* | MNCN8932 | Beira | NA | 1.18 | 0.85 | 0.85 |
| *H. molleri* | IMS4067 | Boceguillas | 0.94 | 0.81 | 1.18 | 1.18 |
| *H. molleri* | IMS4068 | Boceguillas | 0.83 | 0.81 | NA | 1.18 |
| *H. molleri* | IMS4064 | Boceguillas | 0.81 | 0.81 | NA | 1.18 |
| *H. molleri* | IMS4065 | Boceguillas | 0.75 | 0.81 | 1.03 | 1.18 |
| *H. molleri* | IMS4066 | Boceguillas | 0.63 | 0.81 | 1.37 | 1.18 |
| *H. molleri* | IMS4158 | Buenache de la Sierra | 0.62 | 0.64 | 0.78 | 0.96 |
| *H. molleri* | IMS4163 | Buenache de la Sierra | 0.64 | 0.64 | NA | 0.96 |
| *H. molleri* | IMS4164 | Buenache de la Sierra | 0.73 | 0.64 | NA | 0.96 |
| *H. molleri* | IMS4165 | Buenache de la Sierra | 0.67 | 0.64 | NA | 0.96 |
| *H. molleri* | IMS4166 | Buenache de la Sierra | 0.59 | 0.64 | NA | 0.96 |
| *H. molleri* | IMS4157 | Buenache de la Sierra | NA | 0.64 | 0.96 | 0.96 |
| *H. molleri* | IMS4159 | Buenache de la Sierra | NA | 0.64 | 1.08 | 0.96 |
| *H. molleri* | HaT3 | Candeleda | 0.71 | 0.71 | 1.14 | 1.14 |
| *H. molleri* | HaMj1 | Cernégula | 1.03 | 1.03 | 1.17 | 1.17 |
| *H. molleri* | IMS4205 | Codesal - Ferreras de Arriba | 0.56 | 0.84 | NA | 1.18 |
| *H. molleri* | IMS4207 | Codesal - Ferreras de Arriba | 1.11 | 0.84 | NA | 1.18 |
| *H. molleri* | IMS4214 | Codesal - Ferreras de Arriba | NA | 0.84 | 1.17 | 1.18 |
| *H. molleri* | IMS4215 | Codesal - Ferreras de Arriba | NA | 0.84 | 0.99 | 1.18 |
| *H. molleri* | IMS4216 | Codesal - Ferreras de Arriba | NA | 0.84 | 1.18 | 1.18 |
| *H. molleri* | IMS4264 | Codesal - Ferreras de Arriba | NA | 0.84 | 1.55 | 1.18 |
| *H. molleri* | IMS4265 | Codesal - Ferreras de Arriba | NA | 0.84 | 1.08 | 1.18 |
| *H. molleri* | IMS4267 | Codesal - Ferreras de Arriba | NA | 0.84 | 1.18 | 1.18 |
| *H. molleri* | IMS4690 | Cuchía | 0.61 | 0.67 | 0.67 | 0.66 |
| *H. molleri* | IMS4691 | Cuchía | 0.78 | 0.67 | 0.62 | 0.66 |
| *H. molleri* | IMS4692 | Cuchía | 0.81 | 0.67 | 0.61 | 0.66 |
| *H. molleri* | IMS4693 | Cuchía | 0.67 | 0.67 | 0.62 | 0.66 |
| *H. molleri* | IMS4694 | Cuchía | 0.27 | 0.67 | 0.66 | 0.66 |
| *H. molleri* | IMS4695 | Cuchía | NA | 0.67 | 0.75 | 0.66 |
| *H. molleri* | IMS4696 | Cuchía | NA | 0.67 | 0.79 | 0.66 |
| *H. molleri* | IMS4697 | Cuchía | NA | 0.67 | 0.83 | 0.66 |
| *H. molleri* | IMS4698 | Cuchía | NA | 0.67 | 0.46 | 0.66 |
| *H. molleri* | HaGa2 | Embalse de Cecebre | 0.73 | 0.73 | NA | 1.14 |
| *H. molleri* | HaGa1 | Embalse de Cecebre | NA | 0.73 | 1.14 | 1.14 |
| *H. molleri* | IMS3550 | Ginzo de Limia | 0.75 | 0.75 | 1.6 | 0.99 |
| *H. molleri* | IMS3551 | Ginzo de Limia | 0.59 | 0.75 | 0.93 | 0.99 |
| *H. molleri* | IMS3554 | Ginzo de Limia | 1.13 | 0.75 | NA | 0.99 |
| *H. molleri* | IMS3549 | Ginzo de Limia | NA | 0.75 | 0.99 | 0.99 |
| *H. molleri* | IMS1448 | Lavariz | 1.97 | 1.38 | 0.99 | 1.38 |
| *H. molleri* | IMS1449 | Lavariz | 1.38 | 1.38 | 1.48 | 1.38 |
| *H. molleri* | IMS1450 | Lavariz | 1.68 | 1.38 | 1.38 | 1.38 |
| *H. molleri* | IMS1451 | Lavariz | 1.09 | 1.38 | NA | 1.38 |
| *H. molleri* | IMS1452 | Lavariz | 0.76 | 1.38 | NA | 1.38 |
| *H. molleri* | IMS4184 | Longueira | 1.39 | 1.44 | NA | 1.05 |
| *H. molleri* | IMS4177 | Longueira | 1.38 | 1.44 | 1.15 | 1.05 |
| *H. molleri* | IMS4180 | Longueira | 1.51 | 1.44 | NA | 1.05 |
| *H. molleri* | IMS4182 | Longueira | 1.44 | 1.44 | NA | 1.05 |
| *H. molleri* | IMS4183 | Longueira | 1.6 | 1.44 | NA | 1.05 |
| *H. molleri* | IMS4178 | Longueira | NA | 1.44 | 0.95 | 1.05 |
| *H. molleri* | IMS4179 | Longueira | NA | 1.44 | 1.28 | 1.05 |
| *H. molleri* | IMS4185* | Longueira | NA | 1.44 | 0 | 1.05 |
| *H. molleri* | IMS3536B | Monforte de Lemos | 0.82 | 0.82 | NA | 1.25 |
| *H. molleri* | IMS3534B | Monforte de Lemos | NA | 0.82 | 1.09 | 1.25 |
| *H. molleri* | IMS3535 | Monforte de Lemos | NA | 0.82 | 1.51 | 1.25 |
| *H. molleri* | IMS3537 | Monforte de Lemos | NA | 0.82 | 1.25 | 1.25 |
| *H. molleri* | IMS2167 | Navas de Estena - Baños Robledillo | 0.71 | 1.55 | 1.18 | 1.09 |
| *H. molleri* | IMS2170 | Navas de Estena - Baños Robledillo | 1.41 | 1.55 | NA | 1.09 |
| *H. molleri* | IMS2171 | Navas de Estena - Baños Robledillo | 1.7 | 1.55 | NA | 1.09 |
| *H. molleri* | IMS2166 | Navas de Estena - Baños Robledillo | 1.92 | 1.55 | 1.08 | 1.09 |
| *H. molleri* | IMS2168 | Navas de Estena - Baños Robledillo | NA | 1.55 | 0.72 | 1.09 |
| *H. molleri* | IMS4498 | Navas de Estena - Baños Robledillo | NA | 1.55 | 1.11 | 1.09 |
| *H. molleri* | IMS4826 | Ojos de Villaverde | 1.1 | 0.83 | NA | 0.67 |
| *H. molleri* | IMS4828 | Ojos de Villaverde | 0.92 | 0.83 | 0.52 | 0.67 |
| *H. molleri* | IMS4829 | Ojos de Villaverde | 0.87 | 0.83 | NA | 0.67 |
| *H. molleri* | IMS4823 | Ojos de Villaverde | 0.68 | 0.83 | NA | 0.67 |
| *H. molleri* | IMS4824 | Ojos de Villaverde | 0.78 | 0.83 | NA | 0.67 |
| *H. molleri* | IMS4825 | Ojos de Villaverde | 0.26 | 0.83 | NA | 0.67 |
| *H. molleri* | IMS4830 | Ojos de Villaverde | NA | 0.83 | 0.81 | 0.67 |
| *H. molleri* | IMS4839 | Ojos de Villaverde | NA | 0.83 | 0.52 | 0.67 |
| *H. molleri* | IMS4842 | Ojos de Villaverde | NA | 0.83 | 1.08 | 0.67 |
| *H. molleri* | IMS4100 | Ólvega | 1.08 | 0.93 | NA | 0.99 |
| *H. molleri* | IMS4101 | Ólvega | 0.93 | 0.93 | NA | 0.99 |
| *H. molleri* | IMS4102 | Ólvega | 0.88 | 0.93 | NA | 0.99 |
| *H. molleri* | IMS4103 | Ólvega | 1.15 | 0.93 | NA | 0.99 |
| *H. molleri* | IMS4105 | Ólvega | 0.63 | 0.93 | NA | 0.99 |
| *H. molleri* | IMS4097 | Ólvega | NA | 0.93 | 1.08 | 0.99 |
| *H. molleri* | IMS4098 | Ólvega | NA | 0.93 | 0.99 | 0.99 |
| *H. molleri* | IMS4099 | Ólvega | NA | 0.93 | 0.99 | 0.99 |
| *H. molleri* | IMS3542 | Paramos | 1.21 | 1.17 | 1.07 | 1.07 |
| *H. molleri* | IMS3543 | Paramos | 0.85 | 1.17 | 1.05 | 1.07 |
| *H. molleri* | IMS3544 | Paramos | 1.25 | 1.17 | 1.11 | 1.07 |
| *H. molleri* | IMS3545 | Paramos | 1.13 | 1.17 | NA | 1.07 |
| *H. molleri* | IMS3238 | Puerto de la Cubilla | 0.26 | 0.26 | NA | 0.48 |
| *H. molleri* | IMS3235 | Puerto de la Cubilla | NA | 0.26 | 0.74 | 0.48 |
| *H. molleri* | IMS3236 | Puerto de la Cubilla | NA | 0.26 | 0.46 | 0.48 |
| *H. molleri* | IMS3237 | Puerto de la Cubilla | NA | 0.26 | 0.48 | 0.48 |
| *H. molleri* | IMS3932 | Puerto de Urbasa | 0.89 | 0.89 | 0.79 | 0.89 |
| *H. molleri* | IMS3933 | Puerto de Urbasa | 0.59 | 0.89 | 0.89 | 0.89 |
| *H. molleri* | IMS3934 | Puerto de Urbasa | 0.91 | 0.89 | NA | 0.89 |
| *H. molleri* | IMS3931 | Puerto de Urbasa | NA | 0.89 | 1.08 | 0.89 |
| *H. molleri* | IMS4725 | Ruesga | 1.05 | 1.05 | 1.5 | 1.28 |
| *H. molleri* | IMS4726 | Ruesga | 0.86 | 1.05 | 0.79 | 1.28 |
| *H. molleri* | IMS4727 | Ruesga | 1.06 | 1.05 | 1.28 | 1.28 |
| *H. molleri* | IMS4728 | Ruesga | 1.03 | 1.05 | 1.48 | 1.28 |
| *H. molleri* | IMS4729 | Ruesga | 1.07 | 1.05 | 1.25 | 1.28 |
| *H. molleri* | GVA7411 | Santarém | 1.06 | 1.06 | NA | 1.08 |
| *H. molleri* | ERG1275 | Santarém | 0.93 | 1.06 | 1.26 | 1.08 |
| *H. molleri* | GVA7412 | Santarém | 1.25 | 1.06 | NA | 1.08 |
| *H. molleri* | GVA7413 | Santarém | 1.33 | 1.06 | NA | 1.08 |
| *H. molleri* | GVA7414 | Santarém | 0.49 | 1.06 | NA | 1.08 |
| *H. molleri* | ERG1276 | Santarém | NA | 1.06 | 0.94 | 1.08 |
| *H. molleri* | ERG1285 | Santarém | NA | 1.06 | 1.08 | 1.08 |
| *H. molleri* | IMS3556 | Serra da Estrela | 0.87 | 0.92 | 0.99 | 0.99 |
| *H. molleri* | IMS3557 | Serra da Estrela | 0.9 | 0.92 | 0.69 | 0.99 |
| *H. molleri* | IMS3559A | Serra da Estrela | 1.03 | 0.92 | NA | 0.99 |
| *H. molleri* | IMS3555 | Serra da Estrela | 0.95 | 0.92 | 0.69 | 0.99 |
| *H. molleri* | IMS4235 | Serra da Estrela | NA | 0.92 | 1.04 | 0.99 |
| *H. molleri* | IMS4236 | Serra da Estrela | NA | 0.92 | 1.28 | 0.99 |
| *H. molleri* | IMS4237 | Serra da Estrela | NA | 0.92 | 0.99 | 0.99 |
| *H. molleri* | IMS4664 | Sierra de Saceruela | 1.65 | 1.61 | NA | 0.8 |
| *H. molleri* | IMS4666 | Sierra de Saceruela | 0.41 | 1.61 | NA | 0.8 |
| *H. molleri* | IMS4667 | Sierra de Saceruela | 1.84 | 1.61 | NA | 0.8 |
| *H. molleri* | IMS4658 | Sierra de Saceruela | 1.48 | 1.61 | NA | 0.8 |
| *H. molleri* | IMS4659 | Sierra de Saceruela | 1.77 | 1.61 | NA | 0.8 |
| *H. molleri* | IMS4660 | Sierra de Saceruela | 1.58 | 1.61 | NA | 0.8 |
| *H. molleri* | IMS3816 | Sierra de Saceruela | NA | 1.61 | 0.8 | 0.8 |
| *H. molleri* | IMS2925 | Torrefresneda | 0.33 | 0.65 | NA | 0.95 |
| *H. molleri* | IMS2926 | Torrefresneda | 0.96 | 0.65 | NA | 0.95 |
| *H. molleri* | IMS2922 | Torrefresneda | NA | 0.65 | 0.95 | 0.95 |
| *H. molleri* | IMS2923 | Torrefresneda | NA | 0.65 | 1.03 | 0.95 |
| *H. molleri* | IMS2924 | Torrefresneda | NA | 0.65 | 0.79 | 0.95 |
| *H. molleri* | IMS3856 | Valgañón | 0.65 | 0.82 | 1.56 | 1.11 |
| *H. molleri* | IMS3857 | Valgañón | 1.04 | 0.82 | 1.11 | 1.11 |
| *H. molleri* | IMS3858 | Valgañón | 0.73 | 0.82 | 1.09 | 1.11 |
| *H. molleri* | IMS3859 | Valgañón | 0.92 | 0.82 | NA | 1.11 |
| *H. molleri* | IMS4703 | Villaviciosa | 0.48 | 0.55 | 0.88 | 0.89 |
| *H. molleri* | IMS4704 | Villaviciosa | 0.62 | 0.55 | 0.69 | 0.89 |
| *H. molleri* | IMS4701 | Villaviciosa | NA | 0.55 | 0.95 | 0.89 |
| *H. molleri* | IMS4702 | Villaviciosa | NA | 0.55 | 0.89 | 0.89 |
| *H. molleri* | IMS4854 | Villaviciosa | NA | 0.55 | 0.99 | 0.89 |
| *P. cultripes* | PC605 | Aldeaseca de Alba | 0.95 | 0.97 | 1.4 | 1.22 |
| *P. cultripes* | PC606 | Aldeaseca de Alba | 0.99 | 0.97 | 1.05 | 1.22 |
| *P. cultripes* | IMS3888 | Arnedo | 0.88 | 0.91 | 1.22 | 1.31 |
| *P. cultripes* | IMS3890 | Arnedo | 0.93 | 0.91 | 1.4 | 1.31 |
| *P. cultripes* | IMS3062 | Autilla del Pino | 0.58 | 0.59 | 0.35 | 0.44 |
| *P. cultripes* | IMS3067 | Autilla del Pino | 0.6 | 0.59 | 0.52 | 0.44 |
| *P. cultripes* | IMS3163 | Banyeres de Mariola | 0.81 | 0.81 | 0.7 | 0.7 |
| *P. cultripes* | IMS2814 | Bemposta | 0.82 | 0.82 | 0.7 | 0.7 |
| *P. cultripes* | IMS3226 | Bienservida | 1.17 | 1.17 | 1.92 | 1.57 |
| *P. cultripes* | IMS3227 | Bienservida | 1.2 | 1.17 | 1.57 | 1.57 |
| *P. cultripes* | IMS3229 | Bienservida | 1.16 | 1.17 | 1.4 | 1.57 |
| *P. cultripes* | IMS4074 | Boceguillas | 0.93 | 0.94 | 0.7 | 0.7 |
| *P. cultripes* | IMS4075 | Boceguillas | 0.96 | 0.94 | 0.7 | 0.7 |
| *P. cultripes* | PC470 | Boticos | 1.13 | 1.13 | 1.05 | 1.05 |
| *P. cultripes* | PC557 | Burguillos del Cerro | 1.08 | 1.08 | 1.05 | 1.05 |
| *P. cultripes* | IMS3898 | Cabanillas | 0.84 | 0.88 | 1.22 | 1.22 |
| *P. cultripes* | IMS3899 | Cabanillas | 0.91 | 0.88 | 1.22 | 1.22 |
| *P. cultripes* | PC659 | Carpio de Azaba | 0.98 | 0.97 | 1.22 | 1.05 |
| *P. cultripes* | PC660 | Carpio de Azaba | 0.96 | 0.97 | 0.87 | 1.05 |
| *P. cultripes* | PC585 | Don Benito | 1.1 | 1.1 | 1.22 | 1.22 |
| *P. cultripes* | PC700 | Doñana | 1.18 | 1.18 | 1.05 | 1.05 |
| *P. cultripes* | PC701 | Doñana | 1.17 | 1.18 | 1.05 | 1.05 |
| *P. cultripes* | PC702 | Doñana | 1.19 | 1.18 | 1.22 | 1.05 |
| *P. cultripes* | PC406 | El Pedroso | 1.57 | 1.8 | 1.22 | 1.05 |
| *P. cultripes* | PC408 | El Pedroso | 2.02 | 1.8 | 0.87 | 1.05 |
| *P. cultripes* | PC757 | Faro | 1.09 | 1.04 | 1.22 | 1.4 |
| *P. cultripes* | PC758 | Faro | 1.03 | 1.04 | 1.75 | 1.4 |
| *P. cultripes* | PC759 | Faro | 1.04 | 1.04 | 1.4 | 1.4 |
| *P. cultripes* | IMS2294 | Fuendetodos | 1.05 | 1.05 | 0.7 | 0.7 |
| *P. cultripes* | IMS2616 | Gárgoles | 1.15 | 1.1 | 0.7 | 0.79 |
| *P. cultripes* | IMS2626 | Gárgoles | 1.06 | 1.1 | 0.87 | 0.79 |
| *P. cultripes* | IMS3173 | Izagre | 0.79 | 0.79 | 0.52 | 0.79 |
| *P. cultripes* | IMS3174 | Izagre | 0.79 | 0.79 | 1.05 | 0.79 |
| *P. cultripes* | IMS4385 | La Parroquia | 1 | 0.97 | 0.7 | 0.79 |
| *P. cultripes* | IMS4386 | La Parroquia | 0.95 | 0.97 | 0.87 | 0.79 |
| *P. cultripes* | PC846 | Lleida | 0.61 | 0.64 | 0.7 | 0.87 |
| *P. cultripes* | PC848 | Lleida | 0.67 | 0.64 | 1.05 | 0.87 |
| *P. cultripes* | PC941 | Loja | 0.87 | 0.87 | 0.7 | 0.7 |
| *P. cultripes* | PC942 | Loja | 0.91 | 0.87 | 0.7 | 0.7 |
| *P. cultripes* | PC943 | Loja | 0.83 | 0.87 | 0.87 | 0.7 |
| *P. cultripes* | PC377 | Los Escoriales | 1.08 | 1.08 | 0.87 | 0.87 |
| *P. cultripes* | IMS3349 | Los Palancares | 1.11 | 1.97 | 1.75 | 1.49 |
| *P. cultripes* | IMS3351 | Los Palancares | 2.82 | 1.97 | 1.22 | 1.49 |
| *P. cultripes* | PC650 | Malagón | 1.2 | 1.23 | 0.87 | 1.22 |
| *P. cultripes* | PC651 | Malagón | 1.25 | 1.23 | 1.57 | 1.22 |
| *P. cultripes* | PC780 | Malpartida de Cáceres | 1.11 | 1.11 | 1.75 | 1.75 |
| *P. cultripes* | PC628 | Menasalbas | 1.19 | 1.33 | 0.52 | 0.7 |
| *P. cultripes* | PC629 | Menasalbas | 1.33 | 1.33 | 0.7 | 0.7 |
| *P. cultripes* | PC630 | Menasalbas | 1.48 | 1.33 | 1.4 | 0.7 |
| *P. cultripes* | PC885 | Montblanc | 0.28 | 0.28 | 0.52 | 0.52 |
| *P. cultripes* | IMS2612 | Montuenga de Soria | 0.99 | 0.99 | 1.22 | 1.22 |
| *P. cultripes* | IMS2800 | Navalmoral de la Mata | 0.95 | 0.93 | 1.22 | 1.22 |
| *P. cultripes* | IMS2801 | Navalmoral de la Mata | 0.91 | 0.93 | 1.22 | 1.22 |
| *P. cultripes* | PC821 | Noviercas | 1.14 | 1.13 | 1.05 | 0.87 |
| *P. cultripes* | PC822 | Noviercas | 1.13 | 1.13 | 0.7 | 0.87 |
| *P. cultripes* | PC823 | Noviercas | 1.08 | 1.13 | 0.87 | 0.87 |
| *P. cultripes* | PC722 | O Grove | 1.69 | 1.18 | 0.7 | 0.44 |
| *P. cultripes* | PC723 | O Grove | 0.66 | 1.18 | 0.17 | 0.44 |
| *P. cultripes* | PC679 | Paramos | 0.92 | 0.9 | 1.05 | 0.96 |
| *P. cultripes* | PC681 | Paramos | 0.87 | 0.9 | 0.87 | 0.96 |
| *P. cultripes* | IMS3980 | Plage Biscarrosse | 0.12 | 0.13 | 0.17 | 0.26 |
| *P. cultripes* | IMS3981 | Plage Biscarrosse | 0.13 | 0.13 | 0.35 | 0.26 |
| *P. cultripes* | PC737 | Redondo | 1.13 | 1.13 | 1.4 | 1.05 |
| *P. cultripes* | PC738 | Redondo | 1.13 | 1.13 | 0.7 | 1.05 |
| *P. cultripes* | PC739 | Redondo | 1.13 | 1.13 | 1.05 | 1.05 |
| *P. cultripes* | PC865 | Riudarenes | 0.39 | 0.4 | 1.05 | 0.96 |
| *P. cultripes* | PC866 | Riudarenes | 0.4 | 0.4 | 0.87 | 0.96 |
| *P. cultripes* | PC450 | Sedas | 1.25 | 1.19 | 1.05 | 1.22 |
| *P. cultripes* | PC451 | Sedas | 1.13 | 1.19 | 1.4 | 1.22 |
| *P. cultripes* | IMS3329 | Sinarcas | 0.93 | 0.95 | 1.75 | 1.49 |
| *P. cultripes* | IMS3330 | Sinarcas | 0.98 | 0.95 | 1.22 | 1.49 |
| *P. cultripes* | PC926 | Tivissa | 0.62 | 0.62 | 0.35 | 0.35 |
| *P. cultripes* | PC927 | Tivissa | 0.64 | 0.62 | 0.7 | 0.35 |
| *P. cultripes* | PC930 | Tivissa | 0.51 | 0.62 | 0.17 | 0.35 |
| *P. cultripes* | PC800 | Tocha | 0.88 | 0.88 | 0.87 | 0.7 |
| *P. cultripes* | PC801 | Tocha | 0.89 | 0.88 | 0.52 | 0.7 |
| *P. cultripes* | IMS3119 | Valdefinjas | 0.92 | 0.99 | 0.35 | 0.87 |
| *P. cultripes* | IMS3120 | Valdefinjas | 1.06 | 0.99 | 1.4 | 0.87 |
| *P. cultripes* | PC808 | Valencia de Alcántara | 1.09 | 1.09 | 1.22 | 1.22 |
| *P. cultripes* | PC809 | Valencia de Alcántara | 1.09 | 1.09 | 1.22 | 1.22 |
| *P. cultripes* | IMS4045 | Villamayor de los Montes | 0.91 | 0.91 | 0.87 | 0.87 |
| *P. cultripes* | PC394 | Villanueva del Duque | 1.09 | 1.08 | 1.4 | 1.66 |
| *P. cultripes* | PC395 | Villanueva del Duque | 1.07 | 1.08 | 1.92 | 1.66 |

## Figure S1. Sample localities.

_
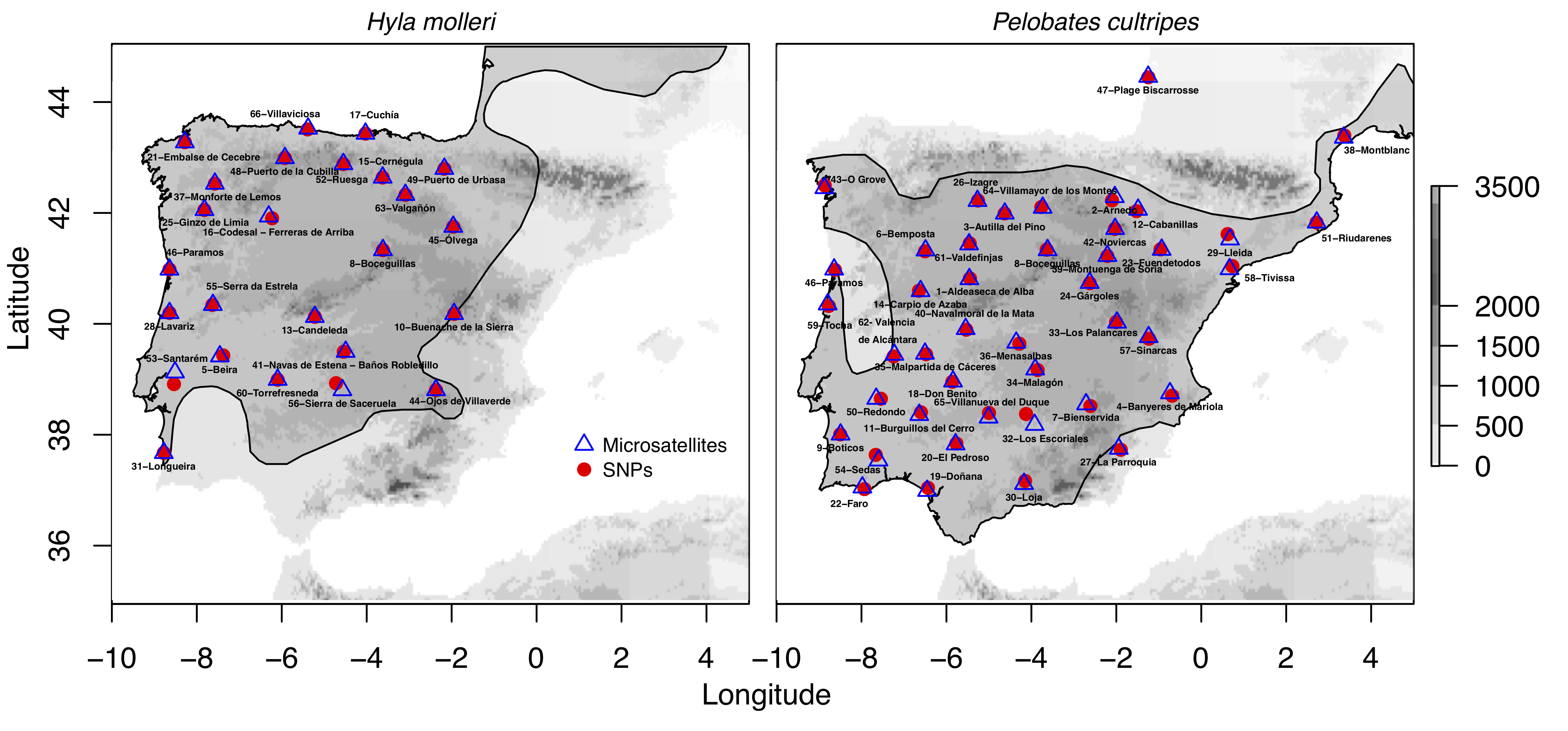
_

Figure S1: Localities sampled for *Hyla molleri* (left panel) and *Pelobates cultripes* (right panel), for microsatellite and SNP genotyping. Coordinates and individual codes are in Table S1. The distribution of each species is indicated by the shaded area (downloaded from the IUCN website on May 2019). For *H. molleri* the map was modified from the distribution of *H. arborea*, because *H. molleri* was recently split from *H. arborea* and its conservation status or distribution have not yet been assessed by the IUCN.

## Figure S2. DArTseq genotype filtering.

Figure S2. SNP filtering for *H. molleri* (A, C, E, G) and *P. cultripes* (B, D, F, H). In each panel we show the frequency distribution (y-axes) of the metric evaluated (x-axes), with the thresholds applied (dashed vertical lines) and individuals/loci retained (green arrow). *Call rate per individual* (threshold 0.35) is the proportion of loci with a call per sample (A, B). *Allele balance* (thresholds 0.15 and 0.85) is proportion of reads for each allele across samples. *Coverage* (threshold 3.5 times larger than the median), is the average coverage of a locus across all samples. *Call rate per locus* (threshold 0.8) is the proportion of samples with a call.

## Figure S3. Likelihood of the STRUCTURE runs.

Figure S3.1. Change of the log likelihood of the STRUCTURE runs from K = 1 to K = 8, for SNPs for *H. molleri.*

Figure S3.2. Change of the log likelihood of the STRUCTURE runs from K = 1 to K = 8, for SNPs for *P. cultripes*.

## Figure S4. Most optimal K.

Figure S4.1. Evolution of the likelihood of the chains in STRUCTURE (P[K|X]), with their corresponding standard deviation from 10 replicate runs for all datasets.

Figure S4.2. Change of Evanno’s ΔK and Parsimony index across K values for all datasets.

## Figure S5. Admixture for STRUCTURE results.

Figure S5. Density plot with absolute differences in Coefficient of Admixture between marker types (|CA_SNPs_ – CA_microsatellites_|) for all individuals of *P. cultripe*s across K. Red values (left side) represent individuals for which admixture with microsatellites was larger than with SNPs, and *vice versa* for blueish values to the right.

## Figure S6. Residuals from genetic diversity.

Figure S6. Residuals from the linear regression sMLH_SNPs_ = sMLH_microsatellites_. Positive values indicate greater sMLH from SNPs compared to sMLH from microsatellites at that given locality for *H. molleri* (A) or *P. cultripes* (B), and negative values indicate the opposite effect.

## Supplementary File S1. Model free hierarchical clustering.

For each dataset, we computed inter-individual Manhattan distances (Kosman & Leonard, 2005) using the *stats::dist* function in R. Every different allele between individuals counted as 0.5 units of distance, and the final distance was corrected by the number of loci evaluated to account for missing data. Clustering was performed applying the NJ algorithm (Saitou & Nei, 1987) on the distance matrices. NJ can describe quite well hierarchical relationships among populations under a wide variety of scenarios (Kalinowski, 2009, 2011; Kopelman, Stone, Gascuel, & Rosenberg, 2013). This model-free approach allows direct comparison for the two marker types (SNPs and microsatellites) under a common reconstruction method, avoiding differences associated with modelling their heterogeneous mutation processes (Di Rienzo et al., 1994; Ellegren, 2004; Valdes et al., 1993; Weber & Wong, 1993; Webster et al., 2002). NJ trees were reconstructed with *njs* function from *ape* 5.0 (Paradis & Schliep, 2018), and bootstrap support values of all internal nodes in the trees were computed with a modified function from *ape::boot.phylo* to accommodate non-standard input data. First, we resampled with replacement loci in the genotype matrices, from which we reconstructed NJ trees. These trees were rooted using midpoint rooting with function *midpoint.root* from *phytools* 0.6-60 (Revell, 2012). Finally, bootstrap values were computed by counting the partitions of the original trees present in 1000 bootstrapped trees.

The clustering topology inferred from the SNP dataset were highly supported at all depths. Conversely, the trees from microsatellites were less supported. For SNPs, the two main groups in the clustering corresponded to the main two groups at K = 2 from STRUCTURE: a northern and a southern lineage for *H. molleri*, and a central-western and a northeastern lineage for *P. cultripes*. Further subdivisions within each major group were highly congruent with clusters retrieved with increasing K values in STRUCTURE. For instance, for the southern clade in *H. molleri* (blue at K = 2), STRUCTURE identified highly nested sub-clustering (up to 6 different main clusters) from K = 2 to K = 8. This nested clustering pattern correlated with long branches in the NJ subgroups (notice the long branch lengths associated with samples from localities from Ojos de Villaverde, id 44, at K4, Serra da Estrela, id 55, at K7, or Longueira, id 31, at K8; Figure SF1.1). However, in clades with more complex branching patterns and shorter branch lengths, STRUCTURE ancestries changed gradually across tips in the tree following correlated geographical and topological clines. For *H. molleri*, this was noticeable, for instance, in the northern clade (orange at K = 2), which showed a cline from “pure” orange eastern localities to increasing magenta ancestries to the west, matching the branching pattern in the tree (Figure SF1.1). For *P. cultripes*, these clines were evident from K = 3 to K = 8 in the central-southern group (blue at K = 2), and mirrored a steady branching topology rather than long branches supporting very differentiated groups. The topologies of the trees inferred from microsatellites were poorly supported, and they were less congruent with STRUCTURE clustering compared to trees from SNPs. As a consequence, clustering built from microsatellite data had less power to support spatial genetic clines or other genetic hierarchical patterns compared to those from SNPs.

**References**

Di Rienzo, A., Peterson, A. C., Garzat, J. C., Valdes, A. M., Slatkint, M., & Freimer, N. B. (1994). Mutational processes of simple-sequence repeat loci in human populations. Proceedings of the National Academy of Sciences of the United States of America, 91(8), 3166–3170. Retrieved from https://www.pnas.org/content/pnas/91/8/3166.full.pdf

Ellegren, H. (2004). Microsatellites: simple sequences with complex evolution. Nature Reviews Genetics, 5(6), 435–445. doi: 10.1038/nrg1348

Kalinowski, S. T. (2009). How well do evolutionary trees describe genetic relationships among populations? Heredity, 102(5), 506–513. doi: 10.1038/hdy.2008.136

Kalinowski, S. T. (2011). The computer program STRUCTURE does not reliably identify the main genetic clusters within species: Simulations and implications for human population structure. Heredity, 106(4), 625–632. doi: 10.1038/hdy.2010.95

Kopelman, N. M., Stone, L., Gascuel, O., & Rosenberg, N. A. (2013). The behavior of admixed populations in neighbor-joining inference of population trees. Pacific Symposium on Biocomputing, 273–284.

Kosman, E., & Leonard, K. J. (2005). Similarity coefficients for molecular markers in studies of genetic relationships between individuals for haploid, diploid, and polyploid species. Molecular Ecology, 14(2), 415–424. doi: 10.1111/j.1365-294X.2005.02416.x

Paradis, E., & Schliep, K. (2018). ape 5.0: an environment for modern phylogenetics and evolutionary analyses in R. Bioinformatics, 35, 526–528.

Revell, L. J. (2012). phytools: an R package for phylogenetic comparative biology (and other things). Methods in Ecology and Evolution, 3(2), 217–223. doi: 10.1111/j.2041-210X.2011.00169.x

Saitou, N., & Nei, M. (1987). The neighbor-joining method: a new method for reconstructing phylogenetic trees. Molecular Biology and Evolution, 4(4), 406–425. doi: 10.1093/oxfordjournals.molbev.a040454

Valdes, A. M., Slatkin, M., & Freimer, N. B. (1993). Allele frequencies at microsatellite loci: The stepwise mutation model revisited. Genetics, 133(3), 737–749.

Weber, J. L., & Wong, C. (1993). Mutation of human short tandem repeats. Human Molecular Genetics, 2(8), 1123–1128. doi: 10.1093/hmg/2.8.1123

Webster, M. T., Smith, N. G. C., & Ellegren, H. (2002). Microsatellite evolution inferred from human-chimpanzee genomic sequence alignments. Proceedings of the National Academy of Sciences of the United States of America, 99(13), 8748–8753. doi: 10.1073/pnas.122067599

Figure SF1.1. Rooted NJ trees for SNP (A, C) and microsatellite (B, D) datasets from *H. molleri* (A, B) and *P. cultripes* (C, D). High/low supported nodes (bootstrap threshold of 700/1000) are depicted by black squares/red circles, respectively. Each tip is identified with a locality ID (Table S1) followed by the individual sample code. Next to them, ancestry proportions from CLUMPAK consensus solutions for the major mode from the 10 replicate runs for K = 2 to K = 8 are shown. Colors in this plot do not necessarily match the clusters from Figure 1.

## Supplementary File S2. Coefficient of admixture

The CA for individual *i* and a given number of clusters *K* (*CA_Ki_*) is the sum of squared ancestry memberships for each *k* cluster (*q_k_*) in that *K*, standardized by the minimum possible value of CA for that K (*CA_Kmin_*; i.e. when memberships are all equal across *k*’s).

- Admixture evenness, $\sum_{1}^{K} q_{ik}^{2}$, is the sum of the squared value of ancestries across clusters for a given K.
- Maximum admixture (A_max_). The maximum possible value of admixture evenness for an individual is that for which the proportions of ancestry are the same between clusters. The $\sum_{1}^{K} q_{ik}^{2}$ term for this individual would be (0.33^2^ + 0.33^2^ + 0.33^2^) = 0.33. Simplified, when admixture is maximum, then:

A_max_ = $\sum_{1}^{K} q_{ik}^{2}$ = 1/*K*

- Coefficient of Admixture:

$${CA}_{Ki}= 1- \frac{\sum_{1}^{K} q_{ik}^{2}- A_{max}}{1 - A_{max}} = \frac{1-\sum_{1}^{K} q_{ik}^{2}}{1 - A_{max}}$$

*CA* oscillates between 0 and 1: 0, indicating all ancestry belonging to a single cluster, and 1, equal proportions across clusters. The code for the functions is available at [github.com/csmiguel/usat_snp](https://www.dropbox.com/s/cilme2py26pv9mw/usat_snp-0.1.0.zip?dl=0)/code/functions/coeff_admixture.r

**Worked example on calculation of CA**

Given the following example matrix of ancestries from STRUCTURE (Q-matrix) for 3 samples (A-C) and 3 genetic clusters (K = 3).

| **Samples** | **k1** | **k2** | **k3** |
| --- | --- | --- | --- |
| **ind_A** | 0.33 | 0.33 | 0.33 |
| **ind_B** | 0.00 | 1.00 | 0.00 |
| **ind_C** | 0.50 | 0.50 | 0.00 |

$$A_{max, K=3}=\frac{1}{3}= 0.33$$

$${CA}_{Ki}= 1- \frac{\sum_{1}^{K} q_{ik}^{2}- A_{max}}{1 - A_{max}} = \frac{1-\sum_{1}^{K} q_{ik}^{2}}{1 - A_{max}}$$

$${CA}_{K=3, ind\_A}= \frac{1-({0.33}^{2} + {0.33}^{2} + {0.33}^{2})}{1 - 0.33}=1.00$$

${CA}_{K=3, ind\_B}= \frac{{1-(0}^{2}+1^{2}+0^{2})}{1 - 0.33}=0$.00

$${CA}_{K=3, ind\_C}= \frac{{1-(0.5}^{2}+{0.5}^{2}+0^{2})}{1 - 0.33}=0.75$$
